# Surgical Delusions and Follies

**Published:** 1885-03

**Authors:** 


**Surgical Delusions and Follies.** By John B. Roberts, A. M., M. D., Professor of Anatomy and Surgery Philadelphia Polyclinic. Philadelphia: P. Blakiston, Son & Co.

This little work is a revision of the address on surgery, for 1884, of the Medical Society of the State of Pennsylvania. It is an attempt to uncover and dispel some surgical superstitions.

